# A rare case report of bilateral vestibulopathy due to otosyphilis

**DOI:** 10.1097/MD.0000000000038149

**Published:** 2024-05-17

**Authors:** Huanhuan Gu, Yixin Xu, Jin Xu, Jianhua Zhuang

**Affiliations:** a Department of Neurology, Second Affiliated Hospital of Naval Medical University, Shanghai, Huangpu District, Shanghai, China.

**Keywords:** bilateral vestibulopathy, otosyphilis

## Abstract

**Rationale:**

Bilateral vestibulopathy is an important cause of imbalance. There are multiple etiologies of bilateral vestibulopathy (BVP), but reports of BVP due to otosyphilis are rare.

**Patient concerns:**

A 39-year-old male was referred to our medical center due to vertigo, persistent dizziness and gait disturbance for 2 months.

**Diagnoses:**

Bilateral vestibulopathy due to otosyphilis was considered in this case, as confirmed through analyses of vestibular function, laboratory tests, and penicillin treatment.

**Interventions:**

The patient was was treated with a high dose of penicillin G (24 × 10^6^ IU/d) for 14 days.

**Outcomes:**

The patient’s symptoms had improved greatly following treatment, with dizziness and gait disturbance having completely resolved at 3 months following hospital discharge.

**Lessons:**

Bilateral vestibulopathy should be considered when evaluating patients with acute or subacute persistent dizziness. Clinicians should also be aware of the potential for otosyphilis among patients who report BVP.

## 1. Introduction

Bilateral vestibulopathy (BVP) is a chronic vestibular syndrome that is characterized by instability while standing or walking together with greater instability, with head movement, in areas with uneven ground, and in dark areas.^[1]^ BVP can develop as a result of a broad spectrum of etiological causes, and 1 analysis of 255 patients revealed that the condition was of unknown etiology in 50% of cases. The most common definite causes include ototoxic drugs, bilateral Meniere’s disease, and infectious causes. Other causes include genetic factors, tumors (bilateral acoustic neuromas, meningeal carcinomatosis), autoimmune diseases (Cogan’s syndrome, nervous system sarcoidosis, Beheet’s disease, cerebral vasculitis), and other rarer causes.^[2]^

Neurosyphilis is a form of chronic infectious disease of the nervous system resulting from infection with *Treponema pallidum.* Upon infection, syphilis can invade the nervous system and result in a wide array of nonspecific symptoms, contributing to a strong potential for misdiagnosis.^[3]^ Otosyphilis is a form of audiovestibular dysfunction due to syphilis,^[4]^ and this condition is very rare. Prior reports pertaining to otosyphilis have primarily focused on hearing impairment, with peripheral vestibulopathy having only been reported only in a few cases.^[5–7]^ The present case details a patient with BVP caused by otosyphilis, and may provide a valuable reference for the diagnosis and clinical treatment of similar patients in the future.

## 2. Case

**Figure 1.**
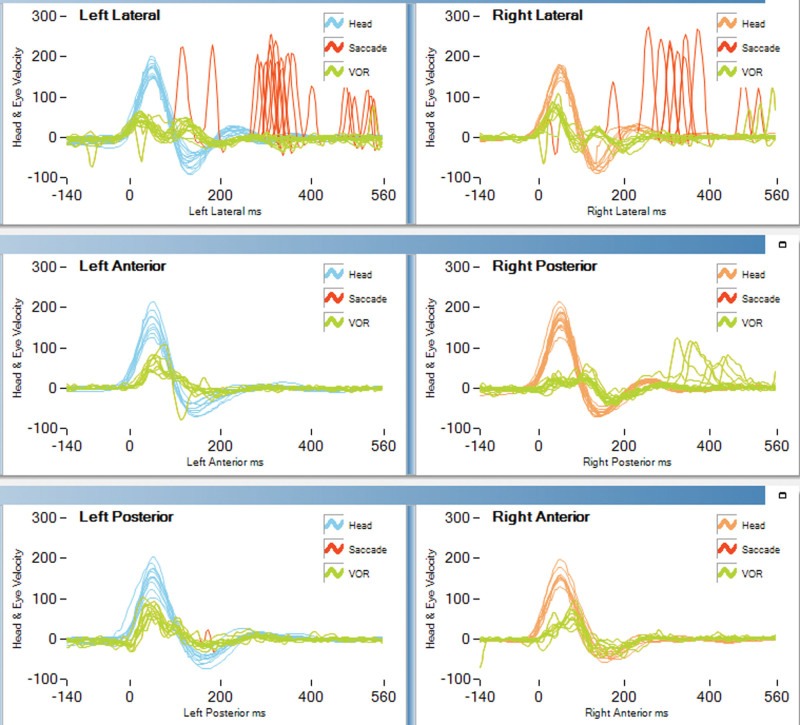
Video head impulse test. The data revealed an obvious decrease in vestibulo-ocular reflex gains in the bilateral horizontal, posterior, and anterior semicircular canals.

**Figure 2.**
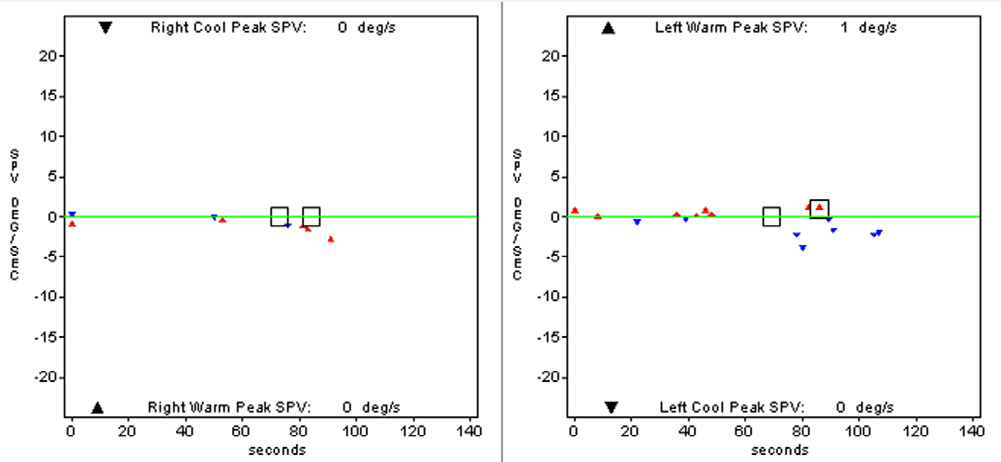
Caloric test results. No response was observed for either caloric test.

A 39-year-old male was referred to our medical center due to vertigo. He presented with persistent dizziness, gait disturbance, nausea, and vomiting for 2 months, and these symptoms were aggravated by dark environments or by walking. He experienced intermittent tinnitus without hearing loss. He denied any history of illness or drug use. Examination revealed corrective scanning in the bilateral head impulse test, while the video head impulse test revealed reduced vestibulo-ocular reflex gains in all 3 bilateral semicircular canals (Fig. 1). No response was observed in either caloric test (Fig. 2), and both cVEMP and oVEMP were absent. Pure-tone audiograms for this patient were largely normal, with no apparent abnormalities having been observed with respect to gaze-evoked nystagmus, pursuit, optokinetic eye movements, or magnetic resonance imaging findings. Based on these results, the patient was diagnosed with BVP. Chemiluminescent assays detected *T. pallidum*-specific antibodies and the TRUST test was positive (TRUST 1:16). Cerebrospinal fluid from this patient exhibited 64 White cells, a protein concentration that was slightly elevated (636 mg/L), TP-CMIA positivity, venereal disease research laboratory test 1:8. The patient was diagnosed with BVP due to syphilis and was treated with a high dose of penicillin G (24 × 10^6^ IU/d) for 14 days. After the course of penicillin treatment, the patient’s dizziness and gait disturbance improved greatly. The patient experienced no nausea and vomiting. Dizziness and gait disturbance had completely resolved, and the head impulse test returned to normal in 3 month follow-up. However, he refused the reexamination of vestibular function and lumbar puncture.

## 3. Discussion

The patient in this case exhibited symptoms and auxiliary examination findings consistent with the diagnosis of BVP. He had a history of good health and was found to be positive for syphilis on laboratory examination. Given the efficacy of penicillin in this case, BVP due to syphilis was considered as a possibility. His symptoms completely resolved at 3 months. Regretfully, he refused to undergo a follow-up test.

Owing to the emergence of antibiotic treatment and the tendency of some patients to intentionally conceal their medical history, rates of syphilis infection have risen in recent years.^[8,9]^ A 2018 report published by the Centers for Disease Control and Prevention report estimated a 71% increase in the rate of early syphilis in the United States from 2014 to 2018, and a growing number of individuals are suffering from complicated syphilis.^[10,11]^ Otosyphilis is 1 potential complication in these patients resulting from the invasion of the inner ear and cranial nerve VIII.

Otosyphilis is generally only described in individual case reports or small clinical trials, making it difficult to establish the true incidence of this condition. Kwananokch described common otosyphilis symptoms in a series of 85 patients, of whom 90.6% had hearing loss, 72.9% had tinnitus, and 52.9% had vertigo.^[12]^ Hearing loss and tinnitus have been described in most prior case reports, whereas vestibular abnormalities have been less frequently described, particularly with respect to nonspecific dizziness. Potential mechanisms of syphilis-associated vestibular cochlear damage include: the direct invasion of the inner ear perilymph, cohleovestibular apparatus, and/or eighth cranial nerve by *T. pallidum*, the spread of spirochetes in the cerebrospinal fluid into the perilymph of the inner ear via the cochlear aqueduct, meningitis caused by *T. pallidum* that affects the 8 cranial nerve, and/or temporal bone and middle ear ossicle involvement in patients with longstanding infections.^[6,13–15]^

While it is recognized, otosyphilis remains a neglected complication of syphilis. It can arise at any stage of the disease and is primarily diagnosed based on clinical symptoms, examinations of vestibular and auditory function, laboratory testing, and the assessment of the efficacy of Benzathine penicillin G.^[4]^ Sometimes referred to as “the great mimicker,” syphilis is a multisystem disease that is readily confused with a range of other conditions. Potential erroneous diagnoses for otosyphilis patients include cochleitis, vestibular neuritis, Meniere’s disease, meningitis, ototoxic drug exposure, cerebrovascular accident, autoimmunity, Cogan’s syndrome, and occupancy diseases.

Treatment strategies for otosyphilis are the same as those employed for neurosyphilis.^[16,17]^ Corticosteroids are recommended in combination with penicillin to suppress inflammation while preventing a Jarisch-Herxheimer reaction, particularly in patients exhibiting disease-related inflammation. During the late stages of otosyphilis, irreversible damage to the cochlea and vestibular systems can occur. When patients are diagnosed and adequately treated at an early time point, however, they have the potential to return to baseline.^[18–20]^

## 4. Limitations

Considering the notable improvement of the patient’s symptoms, painful experience of vestibular function, and the invasion of lumbar puncture, the patient refused to undergo laboratory and assistant examinations in the follow-up period. Evaluation of rehabilitation in the patient were based solely upon symptom improvement and recovery of head impulse test.

## 5. Conclusion

Otosyphilis currently remains a rare cause of BVP that should be actively excluded given the potential for the satisfactory efficacy of early treatment. Clinicians should conduct further inquiries regarding the medical history of patients suffering from persistent dizziness and should perform additional detailed physical examinations, with a particular focus on vestibule and hearing examinations. For bilateral vestibular diseases of unknown origin, particularly among young and middle-aged patients without cerebrovascular disease risk factors, it is important to trace the causes of disease through approaches such as serum-based syphilis screening, thereby reducing misdiagnosis rates to the greatest degree possible.

## Acknowledgments

This work was supported by the National Key Research and Development Program of China (No. 2023YFC2508000 & 2023YFC2508003).

## Author contributions

**Supervision:** Jianhua Zhuang.

**Writing – original draft:** Huanhuan Gu, Yixin Xu.

**Writing – review & editing:** Yixin Xu, Jin Xu, Jianhua Zhuang.
